# Shifting terrains of expertise: Power, knowledge and the era of Industry 4.0

**DOI:** 10.1177/13505084251366177

**Published:** 2026-02-11

**Authors:** Cassandra Bowkett, Christian Lévesque

**Affiliations:** Alliance Manchester Business School, University of Manchester, UK; HEC Montréal, Canada

**Keywords:** AI, expertise, future of work, governance, I.40, knowledge, power

## Abstract

This article examines the social and political construction of Industry 4.0 (I4.0) technologies, situating I4.0 as the latest of several episodes of power that have shaped the shifting terrains of expertise. Expertise and power relations unfold over time, with expert groups exercising persuasive and coercive forms of power through the mobilisation of knowledge amongst other sources. Building on a processual analysis of the Montreal Aerospace cluster, we situate I4.0 as contextually bounded and identify how experts are mobilisers and carriers of knowledge, shifting the terrain of expertise and reproducing and transforming the rules of the game with implications for systemic power relations.

## Introduction

Industry 4.0 (I4.0) is the digitally-driven integration of production using the Internet of things (IoT) to facilitate value creation across all the phases of a product’s lifecycle (Rainnie and Dean, 2020). At its most advanced stages, I4.0 involves the adoption of algorithmic management tools to control production processes. This is achieved via capturing and formatting data, and interconnecting organisation and production systems. To date, adoption rates of I4.0 technologies across countries are low (Krzywdzinski et al., 2022), reflecting an early stage of implementation, and its undetermined trajectory. These technologies are still being constructed and contested in political arenas (Pfeiffer, 2017), and how this process unfolds has significant implications for the future of work, and society more generally (Al-Amoudi, 2023; Deranty et al., 2023).

I4.0 technologies are argued to be reshaping the frontier of knowledge, through extracting and codifying worker knowledge of work processes (Briken, 2020; Moore and Robinson, 2016) and automating both physical and cognitive tasks, thereby reducing worker control over its use and therefore removing a source of worker power. However, knowledge has long produced power, only when mobilised by actors in claims to rationality, and both knowledge and the power it generates are contextually bounded (Flyvbjerg, 1998). Contestation around the frontier of knowledge, and uncertainty have long been the jurisdiction of expert occupations who are able to leverage the indeterminacy of their knowledge to persuade others of their expertise, and of particular courses of action (Larson, 2018; Reed, 1996). Expert authority has been argued to be in crisis, where technological rationalisation and processes of demystification have ‘rendered expert authority as merely a technocratic fixer of the neoliberal regime’ (Reed and Reed, 2022: 3). Important questions emerge about who are the experts, and what expertise and knowledge are valued in the uncertain context of I4.0?

Currently these technologies are being constructed around market rationality, and oriented towards automation and the commodification of human knowledge and experience (Howcroft and Taylor, 2023; Zuboff, 2019, 2022). There is the potential for them to be redirected to serve the broader interests of workers and society, through developing countervailing power, and clear rationale for their application to create better work and to address big societal issues (Acemoglu and Johnson, 2023). Understanding how these technologies could be redirected, requires understanding how their current construction is being shaped by preexisting systemic power relations. We contribute to extant literature by showing that groups of experts are mobilisers and carriers of knowledge that shift the terrain of expertise, playing a central role in the political construction of new technologies. In so doing they reproduce and transform the rules of the game contained within systems of power, with the potential to reshape and redirect the trajectory of I4.0.

Drawing on a 9-year study in the Montreal aerospace industrial cluster we explore the political construction of I4.0 as the latest of three distinct episodes of power (Clegg, 1989), that have transformed who is considered the expert, who’s claims to expertise are legitimised, and who’s knowledge is valued, with implications for systemic power relations. The article proceeds as follows: first, we review the literature on I4.0 and the new frontier of knowledge, then of knowledge, expertise and expert occupations, followed by a discussion on how technology is reshaping systemic power and the terrain of expertise. This is followed by a description of the context and methodology, and the case itself, situating I4.0 into a broader trajectory of the cluster’s development and construction of expertise across three episodes of power. We end with a discussion around how power dynamics, expertise and knowledge within the cluster have transformed the role of expertise and the broader systemic impacts of I4.0, and potential for countervailing power.

## I4.0: Technology and knowledge, the new frontier?

Many of the debates about I4.0 present these technologies as an inevitable form of technologically-driven progress or a technological paradigm, defined by MacKenzie and Wajcman (1999: 13) in its most basic sense ‘as an exemplar’, providing a model for future development. These technologies will herald in a wave of automation, able to replicate human capabilities and therefore replace workers, automating or deskilling their work (Brynjolfsson, 2022; Pfeiffer, 2017). This automation is predicted to occur both in terms of physical and cognitive tasks, for routine work, triggering mass unemployment for lower skilled occupations (e.g. Frey and Osborne, 2013) and via the hollowing out or decomposition of higher skilled professions (Susskind and Susskind, 2015).

These shifts rest on a new frontier of control, workers’ knowledge of the work process: who knows, or has access to this knowledge? I4.0, and AI more generally, are argued to be reshaping the boundaries of knowledge, and who knows, in distinct ways related to the production, construction and interrogation of datasets and databases. Data are produced about the work process, a granular representation of work realities, through processes of knowledge acquisition and production, (Zuboff, 2019) transforming these data into usable information and knowledge for the organisation, a process Zuboff (1988) has termed to ‘informate’. These new forms of value creation rest on the production and interrogation of big data sets, and via the capture, codification and digitisation of workers’ tacit knowledge. Sensors and wearable technologies are able to measure and combine human workers’ movements and reactions and combine them with machine data and existing data sets (Briken, 2020; Moore and Robinson, 2016), while workers have less regulatory protections and capacity to ‘consent’ to these extractions than consumers (Moore, 2024). In essence, worker ‘know-how’ and expertise have the potential to be coopted, and codified. The combination of increased measurement, alongside the algorithm’s capacity to retrieve and analyse such big data sets is argued to result in comprehensive, instantaneous (typically real time), and interactive forms of knowledge (Kellogg et al., 2020).

In so doing, these new technologies have the potential to reshape the ‘indeterminacy of knowledge’ (Reed, 1996; Sewell, 2005). This is where managers cede workers greater discretion to secure their cognitive efforts as a form of control in contexts where it has traditionally been difficult for employers to replicate and/or codify worker knowledge and exert control over the labour process (Sewell, 2005). Where worker tacit (and explicit) knowledge can be captured, there is the potential for reduced worker discretion, and labour power. However, focusing on the dystopian potential of these technologies, and ascribing such power to their materiality can be technologically deterministic. As argued by Burawoy (1979: 87) to secure value-creation employers do not have to control uncertainty entirely. Instead securing worker cooperation requires managing uncertainty, ensuring it is neither too great nor too slight, and that there remains ‘the possibility that workers will assert some control over the labor process, if only of limited kind’.

The reality is that while these technologies have a materiality, they are also socially and politically constructed (MacKenzie and Wajcman, 1999). Technologies are almost always shaped by social power and domination, by ‘designers and interests of power. . . marked by conflict and struggle, and the outcome, therefore is always ultimately indeterminate’ (Noble, 2011: 325). Important questions emerge about how I4.0 is contextually constrained and socially shaped by existing technologies, ‘technical artefacts, institutions and the environment, straddling technical, social, economic and political dimensions’ (Howcroft and Taylor, 2023: 355). Does I4.0 implementation centre automation or augmentation, and are its impacts on work conditions (e.g. labour shedding or work intensification, ibid) contested and resisted? What power struggles emerge over the production of these new bodies of knowledge and who has access to them? How can we understand the degree to which these bodies of knowledge shape who decides, and who controls uncertainty?

## Who decides? Controlling uncertainty, expertise and episodic conflict

I4.0 generates uncertainty, in terms of how these technologies and their extractive processes are reshaping the frontier of knowledge. Experts, and expert occupations have long been understood as central actors in managing uncertainty, organising themselves collectively around knowledge domains, and leveraging the indeterminacy of this knowledge to control its application and use (Freidson, 2001; Reed, 1996). Experts have to navigate a continually shifting balance between the ‘indeterminacy and formalisation of knowledge and skill as a prerequisite to constructing expert power bases and action domains that will stand the test of time’ (Reed, 1996: 578). Which occupations are considered expert professions has been the subject of intense debate (e.g. Fincham, 2012), and jurisdictional struggles over occupational boundaries and knowledge domains (Abbott, 1988) are well documented. The current uncertainty centres on which expert group will be able to mobilise their knowledge to solve the indeterminacy and complexity associated with I4.0 and what power strategies these groups follow to sustain their claims. These battles can be defined as episodes of power (Clegg, 1989) where expert bodies mobilize various sources of power to secure outcomes, such as discursive and narratives resources to shape ideas and sense-making, network resources to build coalition and social capital, and organisational resources to shape rules and decision-making processes (Dorschel, 2021; Greenwood and Suddaby, 2006; Malsch and Gendron, 2013).

Knowledge can be an important source of power, as a form of rationality that is by its very nature contextually rooted (Flyvbjerg, 1998). While knowledge is a source of power, knowledge domains alone are insufficient to establish the expertise of one group over another (Larson, 2018). Expert groups must persuade other actors that this knowledge, and any associated skill and expertise they possess enables them to adequately address the problem or uncertainty at hand, through ‘claims to expertise’ designed to engender collective trust (Reed, 1996), claims that operate as strategies of rationalization (Flyvbjerg, 1998). These claims involve persuasive forms of power, as opposed to more coercive forms of power as domination (Fleming and Spicer, 2014). Successful mobilisation of claims to expertise have historically been the realm of the professions, which ‘emerge from processes of negotiation, conflict and exchange with external stakeholders, such as the state, and with internal competitors’ (Suddaby and Muzio, 2015: 30). These have often rested on justifications of the value of work that operates outside of market principles such as the ‘liberal professions’ (e.g. judiciary, medicine, clergy), with a wider societal role and function, argued by Abbott (1988) to keep the forces of commodification and rationalisation at bay through protections from the state (Reed, 1996).

Experts have traditionally drawn authority from the ‘implicit social contract’ between themselves, the state and the public (Reed and Reed, 2022) where authority is both legitimate and lawful (Spillane and Joullié, 2023) and derived from persuasive forms of power. However expert authority is argued to be in crisis, eroded by broader political and technological shifts (Reed and Reed, 2022), as the occupations who are considered to be experts has changed over time. Broadening to include those with market orientations, experts span a range of traditional (e.g. engineering) and corporate (e.g. HR, consultancy, public relations) professions (Fincham, 2012; Reed, 1996, 2018a), and can include other forms of technical experts such as the skilled trades who share many characteristics with professions, including craft origins and claims to a distinct knowledge domain (Freidson, 2001). In the context of I4.0, it is these latter expert occupations, and actors representing them in the manufacturing industries who are seeking to shape the political construction of the technologies contained within this new production paradigm. The battles or episode of power these groups are engaged in to stake claims to expertise can reinforce, reproduce or transform the rules of the game and the relative power relations between actors, in ways that change the landscape of expertise.

## Technology and systemic power: The changing landscape of expertise

This new era of big data and AI is argued to be characterised by social transformation via division of learning, as opposed to previous organisation via the specialisation or division of labour (Zuboff, 2022). The production and consumption of these new forms of knowledge have the potential to be democratised for the many, or (as they are now) monopolised and commodified for the benefit of the few via surveillance capitalism, in ways that create the conditions for future extreme epistemic inequality (ibid; Zuboff, 2019). These inequalities include the current directionality being challenged, particularly the focus on automation, surveillance, data collection and advertising (Acemoglu and Johnson, 2023: 393), as opposed to how they can augment and improve work and society. A number of authors, including Al-Amoudi (2023) and Pansera and Fressoli (2021) in this journal have also argued for a democratic re-steering of these technologies, harnessing them for the common good, oriented not to economic outcomes such as value creation but to address big societal issues. The importance of redirecting digital technologies away from market driven toward more societally driven aims involves the construction of new rationales (e.g. climate, health, meaning of work), and supporting the building of countervailing power, including worker voices, and collective action as well as the broader interests of citizens and society (Acemoglu and Johnson, 2023). This captures the double movement where market rationality results in a rise in countervailing power (Polanyi, 1957), and the possibility for exchange via reciprocity as opposed to the market (Polanyi, 1974). How can we shape the future of human work both in terms of working conditions to avoid (e.g. powerlessness, boredom, superfluousness) and its broader meaning to society (Deranty et al., 2023)?

Experts will, and already are playing an important role in shaping the directionality of I4.0. Important questions have been asked around what experts are for, and who they are accountable to (Larson, 2018), and these become increasingly salient, as we ask whose expertise is valued in the uncertain political arenas where technology directionality is shaped. The shifting terrain of expert power and role within political systems even prior to these new technological shifts has been well documented, particularly the way that the neoliberal state has eroded democratic mechanisms in favour of what Reed (2018b) refers to as polyarchic “vertically integrated, but streamlined, mechanisms of command and control with a range of horizontally co-ordinated ‘stakeholder co-optation and engagement’ processes through which potentially destabilizing conflicts can be managed” (pp. 16–17). These deliberative mechanisms are populated with technocratic forms of expertise, such as consultants, and financial economic experts representing elite private and corporate interests and supporting particular political interests and policy agendas (Reed, 2018b).

The role of consultants in promoting a specific directionality of I4.0, to shape state policy agendas and investment is well documented (see e.g. Pfeiffer, 2017). Experts play a role in shaping, building and maintaining surveillance and control technologies. When coupled with a neoliberal governmentality, experts may find their expertise subject to processes of demystification, and their authority reduced to ‘technocratic fixers’ (Reed and Reed, 2022). In this dystopian future of expertise and expert authority in crisis, experts ‘frame’ policies and practices, and are responsible for the implementation and maintenance of surveillance regimes but have little autonomy or authority to redirect these technologies’ aims and are often subject to their surveillance effects (Reed and Reed, 2022). Here experts and their expertise are used to ‘rationalise’ (Flyvbjerg, 1998) the directionality of technologies in favour of political actors and a ‘very narrow range of primarily corporate elite, interests and values’ (Reed and Reed, 2022: 8). As these rationalisations (Flyvbjerg, 1998) become more visible, citizens and the broader public loose trust in the objectivity of expertise, eroding the authority of said experts (Flyvbjerg, 1998).

These accounts raise important questions around whether and how these processes of demystification, and the hyper-fixation toward market aims (e.g. automation, surveillance) for digital technologies can be challenged. Can countervailing power be developed to centre the needs of the many (workers, society, the common good), what role does expertise play in this process, and whose’ expertise is valued?

## Context

The global civil aerospace industry is a duopoly centring on Boeing and Airbus, that has emerged from government-owned and funded defence activities in the US and Europe. Challengers to this duopoly include Embraer (Brazil) and Bombardier (Canada) during their forays in civil regional aircraft markets. Aerospace is at the forefront of technological innovation in terms of products, materials, and production processes and is a vital contributor to research and development (R&D) activity. The air force industry, particularly in the US, has played a crucial role in the selection and development of new technology that has spread across the global value chain (Noble, 2011). Governments spanning the global North and South are not only primary purchasers but have also sought to attract and consolidate aerospace manufacturing and innovation activity in their countries. Satellite manufacturing operations with no ties to the innovation system, often referred to as branch plants, are less attractive to governments than those with R&D investment.

Montreal’s aerospace cluster is the world’s third largest centre of aerospace manufacturing (Zhegu, 2007), and cluster intermediary organizations are important actors within political arenas, representing and interceding for the industry. These intermediaries operate as deliberative spaces, enable the voices of firms, trade unions and government representatives to be heard and frame power relations within the cluster. We focus our attention on these organisations as both ‘experts’, and arenas, in the political construction of knowledge as power, expertise and I4.0.

## Methodology

This study of the Montreal aerospace cluster in Quebec, Canada, is part of a long-term research agenda on the aerospace industry, with two phases of data collection, one from 2010 to 2014 (49 interviews) and another from 2015 to 2019 (62 interviews). These were supplemented and triangulated using 42 documents (both internal and public). Semi-structured interviews were conducted with key informants in the cluster’s governance system (regional intermediaries, training and research institutes, cluster associations, government agencies and sectoral unions) and include firm representatives (HR, production and general managers) from firms throughout the supply chain. Interviews, (av. 50 minutes), were recorded, transcribed verbatim and subject to open and thematic coding via NVIVO.

In phase one of the data collection, interviewees were asked about the history of the cluster and evolution of power relations and institutions, as well as key challenges affecting the industry, and the cluster. Challenges included changes in the global industry (e.g. supply chain integration, low-cost competition). Phase 2 of the data collection captures the shift to I4.0, and attempts were made to interview many of the same organisations (and individuals, where feasible) as well as new actors in the changing cluster landscape. These interviews cover the new challenges facing actors, emerging courses of action, and production of resources to support I4.0 implementation. At both phases, interviewees were asked about actor behaviour, and engagement in these processes.

The research design and analysis places power at the core of analysis, starting with a focus on rich detail and ‘little things’, and emphasizing practice before discourse, through a study of context rich cases (Flyvbjerg, 2004). The second author was the principal investigator on the project and has overseen all data collection. This enabled him to gain participants’ trust and included him seeking participant feedback on data analysis at various points, and on outputs of the project (papers, chapters and reports). The authors utilised process analysis practices (e.g. timeline mapping, detailed tables of the actors involved at varying time periods) to move from ‘rich detail and little things’ and identify the practices, projects, and resources produced, the ‘crises’ actors reported, and the claims created at the cluster level to address these crises. The authors moved between these and thematic coding of the interviews to identify three episodes in the shifting terrain of expertise and knowledge which have been used to structure the analysis and contextually situate this latest episode of the political construction of I4.0.

## The evolution of knowledge and expertise in the Montreal aerospace cluster

### Episode 1: Building expertise and a knowledge system (1970–1994)

In the early 1970s, the Montreal Aerospace cluster was a branch plant economy, dominated by subsidiary plants for US companies such as General Dynamics and United Aircraft. The Canadian industry was declining, with employment halving from 48,000 workers in 1967 to 25,000 in 1976 (Zhegu, 2007). The Federal government intervened, stabilising the industry and bailed out the subsidiary of General Dynamics, later bought by Bombardier. During this period firms reported severe skills shortages, in skilled trades (e.g. machinists), technicians, and engineers, resulting in targeted recruiting from abroad, typically England. There were no mechanisms for firms to address labour shortages collectively, and only one dedicated industry training body, École national d’aérotechnique (ÉNA) offering apprenticeship training for skilled trades.

Two major trade unions (IAMAW and UAW) representing the skilled trades played a crucial role in lobbying the federal government to set up joint union-management committees at the sectorial level to solve skill shortages and reduce reliance on foreign recruitment, in part by strengthening the training and knowledge system:

there was a committee set up to see how in Quebec we could better train the workforce, especially reduce foreign recruitment. . . seven or eight hundred people a year, most of whom came from England as welders, assemblers, foremen, engineers, etc. to work in Quebec. The committee identified a human resources problem, described by companies as being the fact that schools were not producing good candidates.

11. Representative, CAMAQ, 2010.

The aerospace joint committee became Comité sectoriel de main-d’œuvre en aérospatiale (CAMAQ) in 1982. CAMAQ operated as a deliberative space, and mediator, in building consensus between the trade unions and HR directors of major firms who were equally represented. The central problem initially identified that later formed CAMAQ’s claim to expertise was that the knowledge system did not produce the right skills, and needed to be strengthened.

Through this period, CAMAQ established itself as a central actor in the cluster, spearheading building the cluster’s pipeline of knowledge development from high school through to master’s level. In 1989 this included establishing a specialist aerospace engineering master’s programme, coordinating across six universities and industry representatives. Following this, CAMAQ led the setup of a specialised trade school (École des métiers de l’aérospatiale de Montréal – ÉNAM), a high school equivalent (16+), offering industry specific trades training:

It’s certain that by having dedicated schools, like the ÉNA, like the ÉMAM, like the joint master’s programme that they initiated, CAMAQ has been able to provide leadership and been able to make the industry heard at the level of initial training 58. Provincial government representative, 2016.

Alongside strengthening knowledge domains and building training systems for several technical and craft occupations, CAMAQ also established an annual labour and skill trends census to anticipate labour shortages. Approximately 80% of firms have continued to contribute to the census, which has been shared on an open-access basis. Sustained participation by firms has provided an accurate picture of labour trends and underlined the willingness of all parties to work together to resolve labour shortages.

Episode 1 is defined less by conflict, more by consensus building, primarily due to CAMAQ’s work as a strategist and boundary spanner, playing a crucial role in developing the knowledge system, identifying problems, and bringing actors together to devise solutions. As the only industry intermediary organisation, CAMAQ shaped outcomes and power dynamics in the cluster through coalition building, with trade union representatives and HR directors at the workplace level. CAMAQ’s claims to expertise rested on skilled craft knowledge, and the need to build a local supply of skilled trades as necessary for the cluster’s growth. This claim was supported by the practical knowledge of HR directors and trade unions representatives of the needs of internal and external labour markets, and rationalized through the knowledge produced by CAMAQ and shared across the cluster on labour market trends. Knowledge and skill were gradually considered as a collective as opposed to private good.

### Episode 2: Competing claims to expertise (1995–2015)

The second episode is characterised by cluster consolidation and restructuring in the mid-2000s around two major aircraft manufacturers Bombardier and Bell Helicopter, a dozen integrators (e.g. Pratt, CAE) and 160 suppliers. Many SME suppliers were skilled machine shops, with expertise built on craft knowledge from the skilled trades, or machinists working for large firms who saw opportunities to take advantage of outsourcing. Employment grew steadily from 23,000 jobs in 1997 to 40,000 in 2015, across existing firms and new subsidiaries of foreign MNCs (Zhegu, 2007). The cluster attracted these firms in part due to its craft knowledge system and strong supply of skilled labour.

This episode also heralds new intermediary organisations. In 1997, an employer association, Association québecoise de l’aérospatiale (AQA) was set up to give a voice to SMEs. Large firms and universities continued to put forward claims to expertise around a need for technical occupations and knowledge, calling for greater provincial state support for research and development. This led to the founding of a research consortium Consortium de recherche et d’innovation en aérospatiale au Québec (CRIAQ) in 2002, supported financially by industry, universities and the state, funding precompetitive innovation research projects between large firms, SMEs and universities. Through CRIAQ, engineers and technical professionals involved in innovation research projects became carriers of expertise by channelling and legitimising their scientific and professional codified knowledge across the supply chain.

The establishment of more large firms as dominant players in the cluster, and proliferation of new intermediary actors resulted in a restructuring of power relations, with Bombardier at the centre. The four largest firms, Bombardier, Bell, Pratt and CAE successfully lobbied local, provincial and federal governments for a cluster organization. In 2006, Aéro-Montréal was set up. Until then CAMAQ was the primary actor addressing industry problems, and trade unions as founders (and the skilled trades they represented) continued to play a central role in shaping the cluster’s agenda.

Aéro-Montréal positioned itself as the main representative of all industry stakeholders interests, including CAMAQ and CRIAQ, to promote dialogue, and ensure a common vision. In reality, Aéro-Montréal was established to represent the interests of large firms in the cluster and liberally utilised their founders as leverage to expand their jurisdiction:

At first it was difficult because when Aéro-Montréal arrived, CRIAQ existed, CAMAQ existed . . . people didn’t like it. Especially since we have the decision makers . . . [and] they do not have these people on their boards. . . .CAMAQ was doing everything it wanted to do for 25 years, and now we’re coming in. . .with CRIAQ, we had the same phenomenon, we created an innovation project. CRIAQ: “But no, innovation is us”. There were some pretty intense discussions at the beginning.

4. Executive Committee Representative, Aéro-Montréal, 2010.

While each intermediary organisation (Aéro-Montréal, CAMAQ, CRIAQ) operated autonomously, developing their own projects, CAMAQ and CRIAQ found their activities restricted to skills and innovation respectively while Aéro-Montréal pushed jurisdictional boundaries to secure strategic control of the cluster. An important component of this shift was how Aéro-Montréal’s leadership prioritised the rationality and claims made by large firms, over the more democratic and industry wide knowledge produced by other expert actors (e.g. CAMAQ), often seeking to undermine or delegitimise its veracity:

CAMAQ will tell you no. Because they do a poll, and then they will tell you, ‘No, there is no [labour or skill] shortage’. When they say that, all the CEOs fall off their chairs. So, that’s right, but they start from a poll they say, ‘there isn’t one, there isn’t one’.

4. Executive committee representative Aéro-Montréal, 2010.

This reordering of jurisdictional boundaries on which expert groups claim expertise was compounded by a concurrent trend of supply chain restructuring, where aircraft manufacturers such as Bombardier were outsourcing more risk to their suppliers. This included innovation capabilities, placing greater onus on Tier 1s and 2s as integrators to manage the supply chain. These pressures together severely undermined the previous craft knowledge regime for both large firms and SMEs and placed SMEs with craft technical knowledge at risk. Their main customers were relocating work to low-cost economies, and they needed to renegotiate their access into supply chains through an integrator.

SME suppliers were expected to set up sophisticated management systems, adopt new production techniques such as ‘lean production’, and engage in partnerships with contractors and manufacturers. Very few rose to the challenge. The expertise of many centred around an entrepreneur, often from a skilled trade with craft but not necessarily managerial, or scientific knowledge. In 2012 Aéro-Montréal absorbed AQA and set up a supply chain working group to internally represent SMEs, making it the main firm representative in the cluster.

The dominant problematisation was that SMEs required support and resources to help them meet these challenges, though their solutions had distinct rationalities. CAMAQ and CRIAQ both adopted more collective solutions. CAMAQ sought to increase collaboration between SMEs though the sharing of good practice between SME managers and through extensive plant tours, while CRIAQ sought to increase SMEs’ technical knowledge through open collaboration research projects. Aéro-Montréal however favoured diffusion of best practice through the standardisation of knowledge generation, leveraging managerial experts such as consultants via the MACH programme.

MACH rested on processes of standardisation, codifying ‘best’ practice and the knowledge associated with it, and developing auditing processes and control mechanisms to ensure that SMEs participating in the programmes were compliant with ‘best practice’ standards for work organisation, human resource, and management practices:

What we wanted with MACH was not to help SMEs to be more productive. What we wanted was to help them to be more competitive. . . Operational management was already their strong suit; but HRM, leadership practices, and governance [fell short].

53. Representative, Aéro-Montréal, 2016

The dominance of best over good practice approaches illustrates the shift within the cluster from craft to standardised forms of knowledge, alongside a focus on innovation and engineering to increase SME competitiveness. The claims to expertise enacted in this episode involve undermining both skilled trades, and craft knowledge that the cluster’s expertise was built on. Instead, greater efforts are placed in engineers, technical occupations and managers, and their bodies of codified knowledge that are easier to standardise and expand through innovation activities.

In this battle over expertise CAMAQ lost ground to Aéro-Montréal. CAMAQ’s director retired in 2014, after a 30-year tenure developing close ties with HR directors and trade union representatives, that formed the heart of CAMAQ’s collaborative coalition building approach. Aéro-Montréal’s claim to expertise rested on the need to strengthen the value chain, particularly SME capabilities, through standardisation and monitoring of best practices. Aéro-Montréal shaped outcomes through control of material and financial resources, and by drawing on the asymmetric power of elite large firms in the cluster and prioritising their interests. This episodic shift to polycentric governance therefore results in the concentration of power.

### Episode 3: Tightening control through I4.0 (2016–2019)

This episode is characterized by a severe uncertainty about the viability of the cluster in a context, where Bombardier was in deep financial difficulty and laid off 6000 employees between 2016 and 2018. Bombardier’s downfall stemmed from overcommitting limited resources, launching three new aircraft models (Learjet 85, Global 7500, CSeries) over a very short period, and trying to compete directly with Boeing and Airbus for the C-Series. Bombardier eventually sold the C-Series to Airbus for 1$ CDN with Quebec Government financial support. Bombardier shifted its focus back to the business jet segment, while Airbus committed to assembling the C-Series in Montreal, securing, to a degree, employment in the cluster, with total employment figures totalling 41,874 in 2019 (CAMAQ, 2023). The arrival of Airbus and several leading suppliers signifies the shift in power from Bombardier to Airbus and broader restructuring of cluster power dynamics.

Aéro-Montréal and several regional partners (e.g. universities) problematised SME difficulties in competing around cost within global industry supply chains, particularly with countries with lower labour costs and positioned adopting I4.0 technologies as the solution, securing government money to do so. This continued the cluster’s previous phase, with one major shift: acknowledgement that none of the cluster’s large firms were themselves ‘experts’ in this new technology.

I4.0 as a policy-driven discourse has been led by Aéro-Montréal and consultancy firms working both independently and with the cluster organization. Not being able to draw on the expertise of large firms with these technologies, Aéro-Montréal has instead coopted and assimilated expert occupations including engineering, technology and management consultants with experience of implementing I4.0 in other sectors, to develop resources to guide firm implementation of these technologies. These consultants are the new experts with Aéro-Montréal leveraging their expertise with digital technologies, data-based decision making, and systemised knowledge, decontextualised from the sector itself. The co-option of external to the sector expertise results in a more techno-centric and deterministic vision of I4.0, prioritising automation of decision making (prescriptive), over other implementation routes which lean towards augmentation.

Aéro-Montréal has successfully positioned itself as the central mediator for I4.0, extending its jurisdictional domain to include both foresight planning on the impact of I4.0 on skill (I4.0 skills guide), and to support SME adoption (MACH FAB 4.0). The strategic orientation of the MACH FAB 4.0 programme was set by Aéro-Montréal and various research centres and funded by government agencies such as the Sous-Traitance Industrielle Québec (STIQ). The programme provided funding, and hired consultants to work with firms, coaching and auditing to support implementation projects spanning real-time production management; optimizing the production cycle using simulation for machine sequencing; data mining for preventive maintenance of manufacturing equipment and interconnecting ERP production systems across the value chain. As of 2019, 37 SMEs were active in the programme, with the objective being to support up to 50 SMEs adopting I4.0.

In addition to MACH FAB 4.0, Aéro-Montréal also tasked the skills working group, including CAMAQ, but primarily led by a consultant to map out I4.0 skills for the industry, producing a skills guide published in 2016. The guide produced outlines a vision of I4.0 predicated on automation and prescriptive data driven decision making, the predicted impacts of these shifts on broad categories of workers and ranks each group’s need for new digital skills. The report predicts that the new experts will be data-based scientific professions, including those engineers who are able to upskill themselves on new digital competencies, supported by technicians (e.g. traditional, programmers). These occupations are the ‘experts’, with only data-based scientific professions identified with the knowledge and skill to train other occupations. Trade occupations (e.g. machinists, assemblers) face a ‘major transformation’, and there is an explicit acknowledgement that the skilled trades will not be upskilled as they are ranked lowest of all occupations for digital skill use, though loss of skill is implicit. The report gives a mixed picture for supervisors, with guidance for up/re skilling in new data skillsets, while also reflecting that:

[Supervisors] could even disappear. This will depend on the quality of the information systems in place. The deployment of real-time information systems and the development of automated planning may well render the role of the supervisor obsolete in the future. The supervisor has a good chance of becoming a virtual system.

Skills Guide, 2016, p. 13

The underlying vision here is one of replacing supervisors with prescriptive algorithmic management tools, and automated data driven decision making, and a reorientation away from trades that have been central in the industry such as machinists. In producing the guide, Aéro-Montréal’s encroachment into CAMAQ’s field of expertise has heightened tensions, and the voice of both CAMAQ and the skilled trades have been significantly reduced, demonstrated by the projected impacts of these technologies on the skills of this once expert technical occupation.

Similarly, via the supply chain working group, MACH FAB 4.0 is being operationalised around best practices identified by consultants, valorising the new data-based production regime, and the automation of decision making. This data-based production regime rests on the acquisition and codification of knowledge around existing manufacturing processes, with a focus on automating and pooling data from a variety of sources (e.g. machines, sensors, clocking, existing software). Huge data sets are then transformed into knowledge using algorithms adopted for both prediction and prescription purposes. While the end goal may be prescription, automated decision making and algorithms as the digital supervisor directing work through a tablet, the human supervisor’s role still exists. However, there is less focus on directing work processes, instead they are responsible for evaluating the effectiveness of, and the decisions made based on these data:

Your supervisor becomes an analyst. . . there is data everywhere, this data must provide information, what do we do with it? We will transform it into a decision-making system, to one day measure: does my system allow me to make good decisions? In the short term, my supervisor takes the data, in 80% of companies, he transforms it into information and in very little time, he will automate a decision.

70. Consultant, 2019

In this I4.0 narrative, where feasible, large data sets need to be created to codify parts of the work process that might previously have required trade and managerial knowledge to undertake. Subsequent testing and implementation of prescriptive algorithmic decision-making can then be used to shift decision-making and knowledge application away from trades and frontline managers and automated under the oversight of senior/centralized management or new ‘expert’ occupations. Supporting this shift, consultants and firms echo the need for new data-based occupations such as data analysts and architects, as much needed but ‘very rare resources’ (79. SME Manager, 2019). These shifts in expert occupations and expertise are justified via a positioning of systemized knowledge generated from ‘big data’ as superior to that of human workers. As suggested by one SME manager involved in MACH FAB: ‘anything you can take away from a human’s decisions, you have to take away from him. . . the human is not good at making decisions’ (77, 2017).

The dominance of this conception of I4.0, which decentres shopfloor and human knowledge in favour of data-centric knowledge, and its deskilling effects on trades such as machinists is visible via accounts from several SMEs participating in MACH FAB who report having changed their recruitment practices. In one such firm, tasks typically done by machinists have been deskilled into operators roles, with this firm reporting reducing the number of its machinists, and trying to recruit low-paid, semi-skilled workers from outside of the industry:

We need the operators, that’s our problem right now. We don’t need people with qualifications. You have a high school diploma, we’ll train you. And we’ll accept people who have not completed high school, and we’ll train them. Because we need more production cell operators, it takes a presence. It takes certain operations to deburr a part, or at any rate to feed the machine, unload it, etc.

103. CEO, SME in MACH FAB 4.0, 2019

This shift from skilled machinists to semi-skilled operators with low skills and low wages represents a form of job degradation, leading to higher turnover rates and new labour shortages. Some trade union representatives discussed how skilled machinists were leaving firms who had implemented such changes, describing their transformed roles as boring and unfulfilling, just ‘changing pellets’. Alongside these shifts are the removal of career development routes as previously supervisors were typically skilled machinists. Now firms are also able to recruit supervisors from outside the industry, to manage ‘by the tablet’, without the necessary knowledge of the manufacturing processes and procedures to engage in problem solving, while decision making about shop floor manufacturing processes are made by engineers or programmers who, in some instances, also do not have sufficient contextual knowledge to foresee issues and problems.

While this ‘mythology’ being generated around big data constructs it as superior to humans enacting their knowledge, the reality is that, oftentimes, the decisions made using the data collected cause new problems and uncertainties. The deskilling of the machinist role into operators has triggered new problems such as labour shortages, triggered by increased numbers of workers leaving, or not entering, the industry because jobs are poor quality, lacking challenge, boring and repetitive. Difficulty in accessing and recruiting new labour for firms in the cluster has put pressure on the skill and training system.

Trade unions and CAMAQ have struggled to contest the dominant conception of I4.0 resting on automation and deskilling. In part their tacit acceptance of the need to adopt I4.0 has created internal conflict within and between unions, weakening CAMAQ’s internal cohesion and reducing its capacity to develop a counter narrative and to build coalitions, which it has traditionally relied on. The delegitimisation of skilled trades and craft knowledge as a form of cluster competitive advantage, exacerbated by the emerging problem of job degradation raise challenges that CAMAQ is struggling to meet.

Aéro-Montréal on the other hand has been able to reinforce its claim to expertise and consolidate its position as the dominant actor, controlling strategic resources, leveraging its elite network and coopting expert occupations to support its strategic aims. The battle around whose knowledge is valued shifts from craft versus standardised and professional (managerial, engineering), to systemised, and data based. The technocratic expert is coopted into Aéro-Montréal’s polyarchic structure to implement its strategic vision of I4.0, and to manage and control any conflict that emerges within the working groups to this vision or associated best practices. This episode is characterised by the deepening and consolidation of power in a handful of actors, through them ‘building digital competence’, and the establishment of governance resting on technocratic expertise that amplifies existing asymmetries of power.

## Discussion

This research sought to explore how experts mobilise knowledge and other sources of power to legitimise and institutionalise their claims to expertise, as a means of controlling technological and organizational uncertainties. In doing so we focus on the gradual transformation of both the boundaries of knowledge that culminated in the deployment of I4.0 and the governance system that evolved from craft to polyarchic forms, coopting experts as technocratic fixers (Reed and Reed, 2022). In contrast to current research on I4.0 that seeks to understand the impact of digitalization on work (Brynjolfsson, 2022; Kellogg et al., 2020), we seek to understand how actors can shape technology upstream before a technology is implemented (Howcroft and Taylor, 2023).

We draw on Clegg’s (1989) framework of power to show changes across various episodes of power in the identity of expert groups, their claims to expertise and the strategies they mobilize to exercise power and deal with indeterminacies. This processual analysis contextualizes and grounds contestation around I4.0 in a broader trajectory of battles around the frontier of knowledge. This analysis also sheds light on how battles over expertise transform and reproduce power relations, the ‘rules of the game’, fixing relations of meaning and membership. We contribute to extant literature by showing that groups of experts are mobilisers and carriers of knowledge that shift the terrain of expertise, playing a central role in the political construction of new technologies. In so doing they reproduce and transform the rules of the game contained within systems of power with the potential to reshape and redirect the trajectory of I 4.0.

## Shifting terrains and battles of expertise across episodes of power

Table 1 outlines how knowledge and expertise evolve across the three episodes of power, and the transformation of power dynamics between experts and dominant expert organisations.

**Table 1.** Episodes of power.

Time period	Episode 1	Episode 2	Episode 3
EXPERT	Skilled trades, engineers	Engineers, managers	Consultants, data-centric occupations
KNOWLEDGE	Craft	Standardisation	Systemisation
CLAIMS TO EXPERTISE	Need to build local supply of skilled trades	Need to upgrade SME capabilities	Need to build digital competence
DOMINANT EXPERT ORGANISATION	CAMAQ	Aéro-Montréal; CAMAQ; CRIAQ	Aéro-Montréal
PROBLEMATISATION	Production of knowledge and skill as collective goods	Diffusion and sharing of best practices	Commodification of expertise
POWER DYNAMICS			
FORMS	Persuasion	Persuasion and coercion	Coercion
SOURCES	Networking and social capital	Control over resources, position in social and organisational networks + position in supply chain network (authority)	Control over resources leveraging elite networks

During the first episode, CAMAQ positioned itself as the central expert organization controlling uncertainty associated with the problem of labor shortages. CAMAQ’s claim to expertise rested on a craft narrative around the need to build local supplies of skilled labor, and creating resources to track labor market skill trends and anticipate future skill needs. CAMAQ operated as a boundary spanner (Greenwood and Suddaby, 2006) bringing actors together and creating a shared narrative at the cluster level around the need to expand and strengthen the cluster’s knowledge system. Using loose deliberative processes, it persuaded actors to problematise knowledge/skill as a collective good produced and shared by all stakeholders. In this first episode of power, CAMAQ’s sources of power rely on persuasion, via coalition building and social capital developed in the longue durée (Flyvbjerg, 1998).

Episode two is characterized by the establishment of polyarchic governance built around new expert organizations, CRIAQ and Aéro-Montréal. During this episode, Aéro-Montréal sought to expand the boundaries of its jurisdiction and constrain CAMAQ and CRIAQ’s knowledge domains, leading to jurisdictional conflicts and a battle over claims to expertise. Aero-Montréal positioned itself as the expert of experts, attempting to delegitimize CAMAQ’s claims to expertise by leveraging the authority of the CEOs on its board. This battle is evident in the distinct problematisations of how to upgrade SME capabilities and strengthen the value chain. CAMAQ and Aéro-Montréal agreed the solution involved diffusing best practices but disagreed on how to achieve this. CAMAQ adopted a persuasive and collaborative approach of ‘learning by doing (and showing)’. Aéro-Montréal promoted monitoring and standardizing processes utilising management consultants. Aéro-Montréal was able to prevail through coercion, exercising control over material and financial resources and leveraging ‘shadow’ power from its elite corporate member firms. This enabled it to position itself as the actor and deliberative space where cluster strategy on key issues was decided. In so doing, it was able to use its polyarchic structure to create both vertical command and control, deciding the cluster strategy that prioritized the needs of large firms, and using horizontal ‘stakeholder co-option and engagement’ processes (Reed, 2018b) via its working groups to try and restrict ‘conflicts’. Here the stage is set for consolidation of power via technocratic governance during the third episode.

By the third episode Aéro-Montréal has established itself as the voice of large firms and engaged in a number of conflicts to expand its jurisdiction. With the introduction of I4.0 Aéro-Montréal’s encroachment into CAMAQ’s field of expertise is complete, as it takes responsibility for strategically outlining the future skills needed for this new production paradigm. This hostile renegotiation of boundaries has resulted in Aéro-Montréal positioning itself as the center for digital expertise and reinforced its claim of the need to build digital competence. Aéro-Montréal now controls strategic resources, leveraging the expertise of elite networks and consultants whose knowledge enables the conception and adoption of I4.0 as technocratic experts within the polyarchic governance structure (Reed, 2018b) to manage specific projects, and defer to Aéro-Montréal’s strategic vision of I4.0.

Aéro-Montréal becomes ‘a framer of policies and programmes, as well as constructing the knowledge system and organizational technologies whereby they can be rolled out’ (Reed and Reed, 2022: 7), and the battle around what knowledge is valued thus shifts to systematised and databased. The impacts of this tightening of control are starting to emerge, through the transformation of work, indicative of the hollowing out and deskilling of the skilled trades, via the extraction of their knowledge (Noble, 2011; Susskind and Susskind, 2015). This degradation of work represents reduced autonomy and increased boredom, superfluousness and powerlessness (Deranty et al., 2023), and early signs suggest that this may be causing a new problem: the strict application of systematised knowledge by technocratic experts creates and expands the shortage of labour via degradation of the quality of work.

Our analysis of these three episodes of power in the shaping of I4.0 has three implications. First, we argue that the process by which boundaries of knowledge are reshaped from craft to standardized and then systematised are constituted around the strategic action of various occupational and expert groups over time: skilled trades, managers and engineers, and consultants and data-centric occupations. Here, knowledge and claims to expertise are context dependent (Flyvbjerg, 1998) and reflect capacity of expert groups to address contextually bounded ‘problems’ and cope with related uncertainties. These shifts lead to a form of rationalization that favours universalistic and context independent problematisation, a form of decontextualized best practice.

Second, we highlight that there is a gradual shift in the sources and forms of power mobilised and exercised by experts. While knowledge is a source of power (Flyvbjerg, 1998), knowledge remains insufficient for a group of experts to establish expertise (Larson, 2018). Groups of experts need to mobilise other power sources, such as discursive and narrative, network and organisational, and material resources (Dorschel, 2021; Greenwood and Suddaby, 2006; Malsh and Gendron, 2013). Expert organisations may be able to leverage traditional forms of expert authority that are persuasive in nature, alongside more coercive forms of power, as is the case with Aéro-Montréal. In our case, evidence suggests that rationality and more persuasive forms of power can struggle to form effective countervailing power (Acemoglu and Johnson, 2023) against Aéro-Montréal’s more coercive ‘shadow’ power, which rests on existing industry asymmetries. Third, the diversification of groups of experts via polycentric governance has not led to a corresponding diversification but to a concentration of power in the hands of a small core of political and corporate elite (Reed, 2018b). Expert occupations, particularly consultants, become technocratic fixers (Reed and Reed, 2022) channeling best practices associated with I4.0 and reinforcing the systematization of knowledge. Initially, expert groups problematized knowledge predominantly as a common good produced and shared by all stakeholders through reciprocity exchange (Polanyi, 1974). However, gradually, knowledge has come to be problematized as a market commodity based on asymmetric exchange (Zuboff, 2019, 2022).

## I4.0 expertise and the transformation/reproduction of systemic power relations

As we move from analysing the shifting terrains of expertise across our three episodes of power, to examining systemic power relations (Clegg, 1989) we focus on the continuous tension between reproduction and transformation, and how these tensions are reshaping the rules of the game, and the degree of uncertainty and indeterminacy that remain (Burawoy, 1979; Reed, 1996; Sewell, 2005). Systemic power shifts occurring between episodes one and two involve creating ‘rules of the game’ as expert organizations leverage the knowledge of expert occupations to reduce and control uncertainty. These rules enable actors within the cluster to address skill shortages, first among the skilled trades, and second among SME managers. They involve various experts and expert organisations who leverage their knowledge to control the uncertainty the cluster faces (Reed, 1996; Sewell, 2005).

Between episodes two and three we see a reproduction via consolidation of these rules, and by proxy the consolidation and deepening of power asymmetries in the hands of corporate and political elite (Reed, 2018b). It is in this consolidation that we see a transformation of systemic power relations via the process of demystification (Reed and Reed, 2022). As power becomes concentrated in and around Aero-Montreal and the elite interests it represents, they exert greater control over the efforts of other expert organisations, and the select expert occupations they have coopted. These experts act as technocratic fixers of these systems, even as their expert authority is demystified, as their co-option and alignment with elite interests becomes more visible (Reed, 2018b; Reed and Reed, 2022).

In this context I4.0 is constructed as a tool for automation, surveillance and data extraction, aligned to market rationalities (Acemoglu and Johnson, 2023; Brynjolfsson, 2022), and is used to control uncertainty via extracting worker knowledge and separating the production of worker knowledge from its consumption (Zuboff, 2022). In this process, workers lose some of the indeterminacy in their work, (Briken, 2020; Moore and Robinson, 2016), while experts lose their autonomy and expert authority (Reed and Reed, 2022). This tightening of control over the degree of uncertainty paradoxically results in the increasing fragility of these systems of power. The collapse of what Acemoglu and Johnson (2023) call countervailing power places the future of the cluster into question.

What we see starting to emerge from this tightening of control over uncertainty is the possibility of a double movement (Polanyi, 1957). The degradation of the machinist role triggers new labour and skill shortages, as workers seek more meaningful work elsewhere. The weakening of CAMAQ triggers the possibility of how it can be rebuilt and repositioned, with the potential for contestation and resistance. In essence a certain degree of uncertainty is necessary to ensure actors consent to the broader rules of the game (Burawoy, 1979), and here as this uncertainty is reduced, the potential for contestation and resistance increases, and with this the possibility to redirect these technologies.

## Conclusion

To redirect I4.0 and other post-human technologies (Al-Amoudi, 2023) we need to support the development of countervailing power formed from a constellation of worker, citizen and expert groups, organized around directionality other than market rationality (Acemoglu and Johnson, 2023). Our work has argued the necessity of acting upstream, to try and shape how technologies are politically constructed, and highlighted the central role expert intermediary organizations with expertise in skill deployment and technology can play in this process. Actors within such constellations need to build their strategies in ways that centre important philosophical questions and debates about the meaning and future of work, and of society. For workers and worker representatives this includes shifting strategies to discuss both job quantity and quality, and the latter should be of interest to both policymakers and employers. These technologies, and their systemic data could be democratised to solve the big problems facing society today (Acemoglu and Johnson, 2023; Al-Amoudi, 2023; Zuboff, 2019). Doing so requires active work challenging their extractive application, and the commodification as opposed to democratization of work and human knowledge. Experts, and expert organizations have a crucial role to play in these episodes of power and are well placed to either usher in a new technocratic regime, or to join movements of countervailing power to resist and redirect it, with implications for how systemic power relations are reshaped and/or consolidated.

However, given the contextual nature of power, as both episodic and systemic (Clegg, 1989), one limitation of this study is the specificity of the Montreal aerospace industry. The dynamics of power relations, particularly that of the corporate elite (e.g. control of large firms over suppliers) and countervailing power (e.g. worker power, routine occupations versus skilled trades) may vary and be more fragmented in other manufacturing industries and regions, and this may shape the capacity for actors, both workers and firms, to collectively organise. These varying constellations of power are likely to shape the emergence and construction of I4.0, and future research could examine these variations, and contrast them with their technological counterparts in other industries.

## References

[bibr1-13505084251366177] Abbott Andrew . (1988) The System of Professions: An Essay on the Division of Expert Labor. Chicago, IL: University of Chicago press.

[bibr2-13505084251366177] Acemoglu Daron Johnson Simon (2023) Power and Progress: Our Thousand-Year Struggle Over Technology and Prosperity. London: Basic Books UK.

[bibr3-13505084251366177] Al-Amoudi Ismael . (2023) ‘The Politics of Post-Human Technologies: Human Enhancements, Artificial Intelligence and Virtual Reality’, Organization 30(6): 1238–45.

[bibr4-13505084251366177] Briken Kendra (2020) ‘Welcome in the Machine: Human–Machine Relations and Knowledge Capture’, Capital & Class 44(2): 159–71.

[bibr5-13505084251366177] Brynjolfsson Erik (2022) The Turing Trap: The Promise & Peril of Human-Like Artificial Intelligence. Stanford, CA: Stanford Digital Economy Lab; Dædalus.

[bibr6-13505084251366177] Burawoy Michael (1979) Manufacturing Consent. Chicago, IL: University of Chicago.

[bibr7-13505084251366177] CAMAQ, Comité sectoriel de main-d’oeuvre en aérospatiale au Québec (2023) Recensement des emplois au 1er Janvier 2022, et prévisions des emplois aux 1er Janvier 2023 et 2024. Industrie de la fabrication aéronautique et spatiale au Québec. Montreal: CAMAQ.

[bibr8-13505084251366177] Clegg Stewart (1989) Frameworks of Power. London: Sage Publications.

[bibr9-13505084251366177] Deranty Jean-Philippe Rhodes Carl Yeoman Ruth (2023) ‘Does Work have a Future? The Need for New Meanings and New Valuings of Work’, Organization 30(5): 799–808.

[bibr10-13505084251366177] Dorschel Robert (2021) ‘Discovering Needs for Digital Capitalism: The Hybrid Profession of Data Science’, Big Data & Society 8(2): 1–13.

[bibr11-13505084251366177] Fincham Robin (2012) ‘Expert Labour as a Differentiated Category: Power, Knowledge and Organisation’, New Technology, Work and Employment 27(3): 208–23.

[bibr12-13505084251366177] Fleming Peter Spicer André (2014) ‘Power in Management and Organization Science’, Academy of Management Annals 8(1): 237–98.

[bibr13-13505084251366177] Flyvbjerg Bent (1998) Rationality and Power: Democracy in Practice. Chicago, IL: University of Chicago Press.

[bibr14-13505084251366177] Flyvbjerg Bent (2004) ‘Phronetic Planning Research: Theoretical and Methodological Reflections’, Planning Theory & Practice 5(3): 283–306.

[bibr15-13505084251366177] Freidson Eliot (2001) Professionalism: The Third Logic. Cambridge: Polity Press.

[bibr16-13505084251366177] Frey Carl Benedikt Osborne Michael . (2013) The Future of Employment. Oxford: University of Oxford.

[bibr17-13505084251366177] Greenwood Royston Suddaby Roy (2006) ‘Institutional Entrepreneurship in Mature Fields: The Big Five Accounting Firms’, Academy of Management Journal 49(1): 27–48.

[bibr18-13505084251366177] Howcroft Debra Taylor Phil (2023) ‘Automation and the Future of Work: A Social Shaping of Technology Approach’, New Technology, Work and Employment 38(2): 351–70.

[bibr19-13505084251366177] Kellogg Katherine C Valentine Melissa A Christin Angele (2020) ‘Algorithms at Work: The New Contested Terrain of Control’, Academy of Management Annals 14(1): 366–410.

[bibr20-13505084251366177] Krzywdzinski Martin Gerst Detlef Butollo Florian (2022) ‘Promoting Human-Centred AI in the Workplace. Trade Unions and their Strategies for Regulating the Use of AI in Germany’, Transfer: European Review of Labour and Research 29(1): 53–70.

[bibr21-13505084251366177] Larson Magali Sarfatti . (2018) ‘Professions Today: Self-Criticism and Reflections for the Future’, Sociologia, Problemas e Práticas 88: 27–42.

[bibr22-13505084251366177] MacKenzie Donald Wajcman Judy (1999) The Social Shaping of Technology. Buckingham: Open University Press.

[bibr23-13505084251366177] Malsch Bertrand Gendron Yves (2013) ‘Re-Theorizing Change: Institutional Experimentation and the Struggle for Domination in the Field of Public Accounting’, Journal of Management Studies 50(5): 870–99.

[bibr24-13505084251366177] Moore Phoebe Robinson Andrew (2016) ‘The Quantified Self: What Counts in the Neoliberal Workplace’, New Media & Society 18(11): 2774–92.

[bibr25-13505084251366177] Moore Phoebe V (2024) ‘Workers’ Right to the Subject: The Social Relations of Data Production’, Convergence 30(3): 1076–98.

[bibr26-13505084251366177] Noble David (2011) Forces of Production: A Social History of Industrial Automation. New Jersey: Transaction Publishers.

[bibr27-13505084251366177] Pansera Mario Fressoli Mariano (2021) ‘Innovation Without Growth: Frameworks for Understanding Technological Change in a Post-Growth Era’, Organization 28(3): 380–404.

[bibr28-13505084251366177] Pfeiffer Sabine (2017) “The Vision of ‘Industrie 4.0’ in the Making—A Case of Future Told, Tamed, and Traded’, Nanoethics 11(1): 107–21. 10.1007/s11569-016-0280-3 PMC5383681 28435474

[bibr29-13505084251366177] Polanyi Karl (1957) The Great Transformation. Massachusetts: Beacon Press.

[bibr30-13505084251366177] Polanyi Karl (1974) ‘L’économie en Tant Que Procès Institutionnalisé’, in Gaudelier M (ed) Un Domaine Contesté: L’anthropologie Économique, pp. 153–81. Paris: De Gruyter Mouton.

[bibr31-13505084251366177] Rainnie Al Dean Mark (2020) ‘Industry 4.0 and the Future of Quality Work in the Global Digital Economy’, Labour & Industry: A Journal of the Social and Economic Relations of Work 30(1): 16–33.

[bibr32-13505084251366177] Reed Cara (2018a) ‘Professionalizing Corporate Professions: Professionalization as Identity Project’, Management Learning 49(2): 222–38.

[bibr33-13505084251366177] Reed Cara Reed Michael (2022) ‘Expert Authority in Crisis: Making Authority Real Through Struggle’, Organization Theory 3(4): 1–21.

[bibr34-13505084251366177] Reed Michael I (1996) ‘Expert Power and Control in Late Modernity: An Empirical Review and Theoretical Synthesis’, Organization Studies 17(4): 573–97.

[bibr35-13505084251366177] Reed Michael I (2018b) ‘Elites, Professions, and the Neoliberal State: Critical Points of Intersection and Contention’, Journal of Professions and Organization 5(3): 297–312.

[bibr36-13505084251366177] Sewell Graham (2005) ‘Nice Work? Rethinking Managerial Control in an Era of Knowledge Work’, Organization 12(5): 685–704.

[bibr37-13505084251366177] Spillane Robert Joullié Jean-Etienne (2023) ‘The Decline of Authority and the Rise of Managerialism’, Organization 30(5): 961–80.

[bibr38-13505084251366177] Suddaby Roy Muzio Daniel (2015) ‘Theoretical Perspectives on the Professions’, In: Empson L Muzio D Broschak J Hinings. B (eds) The Oxford Handbook of Professional Service Firms, p. 47. Oxford: Oxford University Press.

[bibr39-13505084251366177] Susskind Richard E Susskind Daniel (2015) The Future of the Professions: How Technology Will Transform the Work of Human Experts. Oxford: Oxford University Press.

[bibr40-13505084251366177] Zhegu Majlinda (2007) La Coévolution des Industries et des Systèmes d’innovation: L’Industrie Aéronautique. Montréal: Montréal Université du Québec à Montréal.

[bibr41-13505084251366177] Zuboff Shoshana (1988) In the Age of the Smart Machine: The Future of Work and Power. New York, NY: Basic Books.

[bibr42-13505084251366177] Zuboff Shoshana . (2019) The Age of Surveillance Capitalism: The Fight for a Human Future at the New Frontier of Power. New York: Public Affairs.

[bibr43-13505084251366177] Zuboff Shoshana (2022) ‘Surveillance Capitalism or Democracy? The Death Match of Institutional Orders and the Politics of Knowledge in Our Information Civilization’, Organization Theory 3(3): 1–79.

